# Supplementary material for: Genetic regulation of RNA splicing in human pancreatic islets
Source: Genome Biol. 2022 Sep 15;23:196. doi: 10.1186/s13059-022-02757-0 (PMC9479353; doi:10.1186/s13059-022-02757-0)
Supplement: Supplementary file 1 — Additional file 1: Supplementary Figures S1-S9 and figure legends. [file 13059_2022_2757_MOESM1_ESM.pdf]

Fig. S1

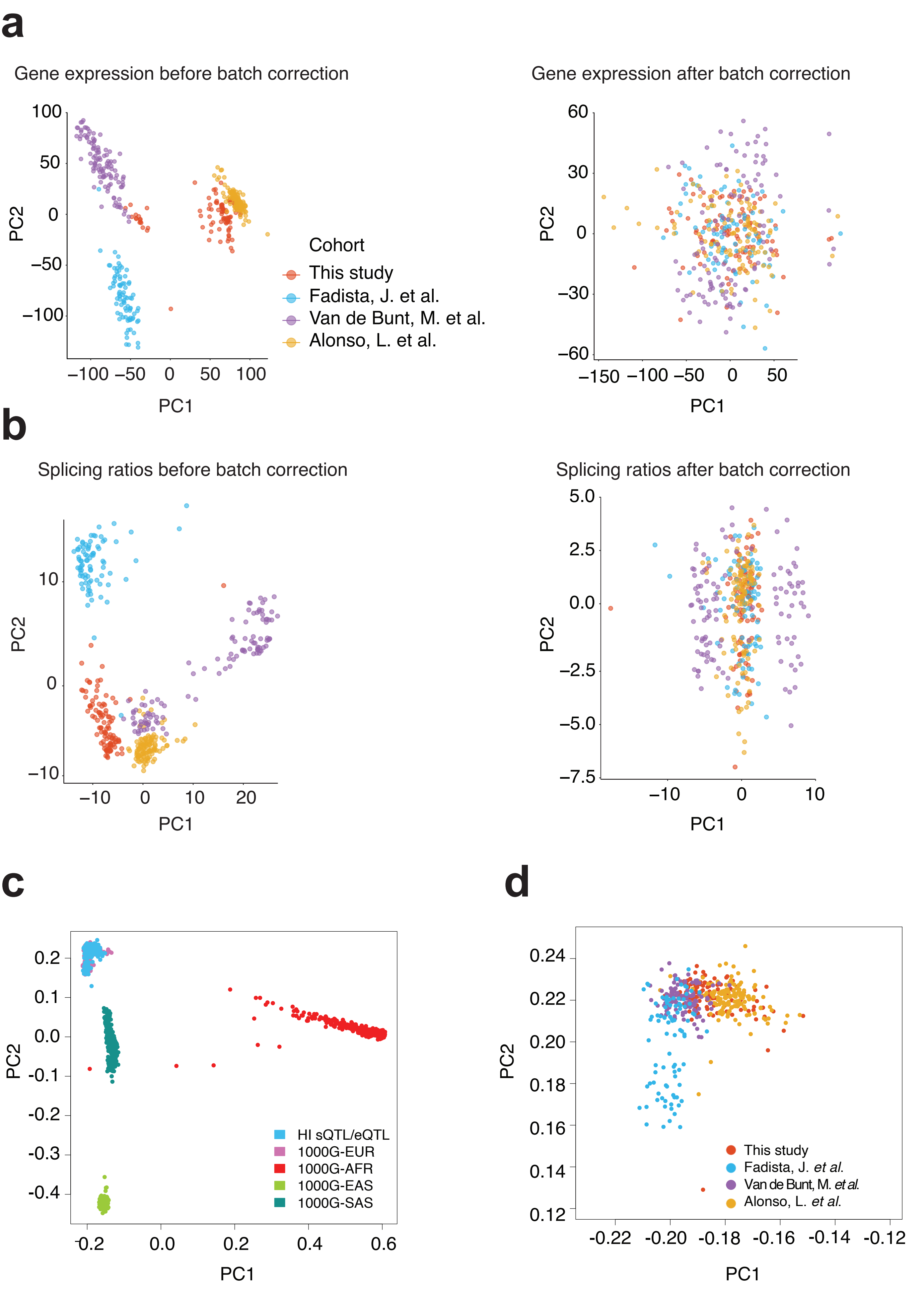

Figure S1. Principal component (PC) analysis based on RNA-seq and genotype data in 399 qualifying human islet samples. A-B PCs calculated using gene expression and *Leafcutter* junction usage profiles before and after correcting for batch effects. **C** PCs calculated from genotypes. Islet sample donors (light blue) were positioned according to PC1 (x-axis) and PC2 (y-axis) calculated from 1000 Genomes Phase 3 genotypes. **D** Differences in population structure according to genetic PC1 (x-axis) and PC2 (y-axis) between the four cohorts included in our panel of 399 islet transcriptomes.

Fig. S2

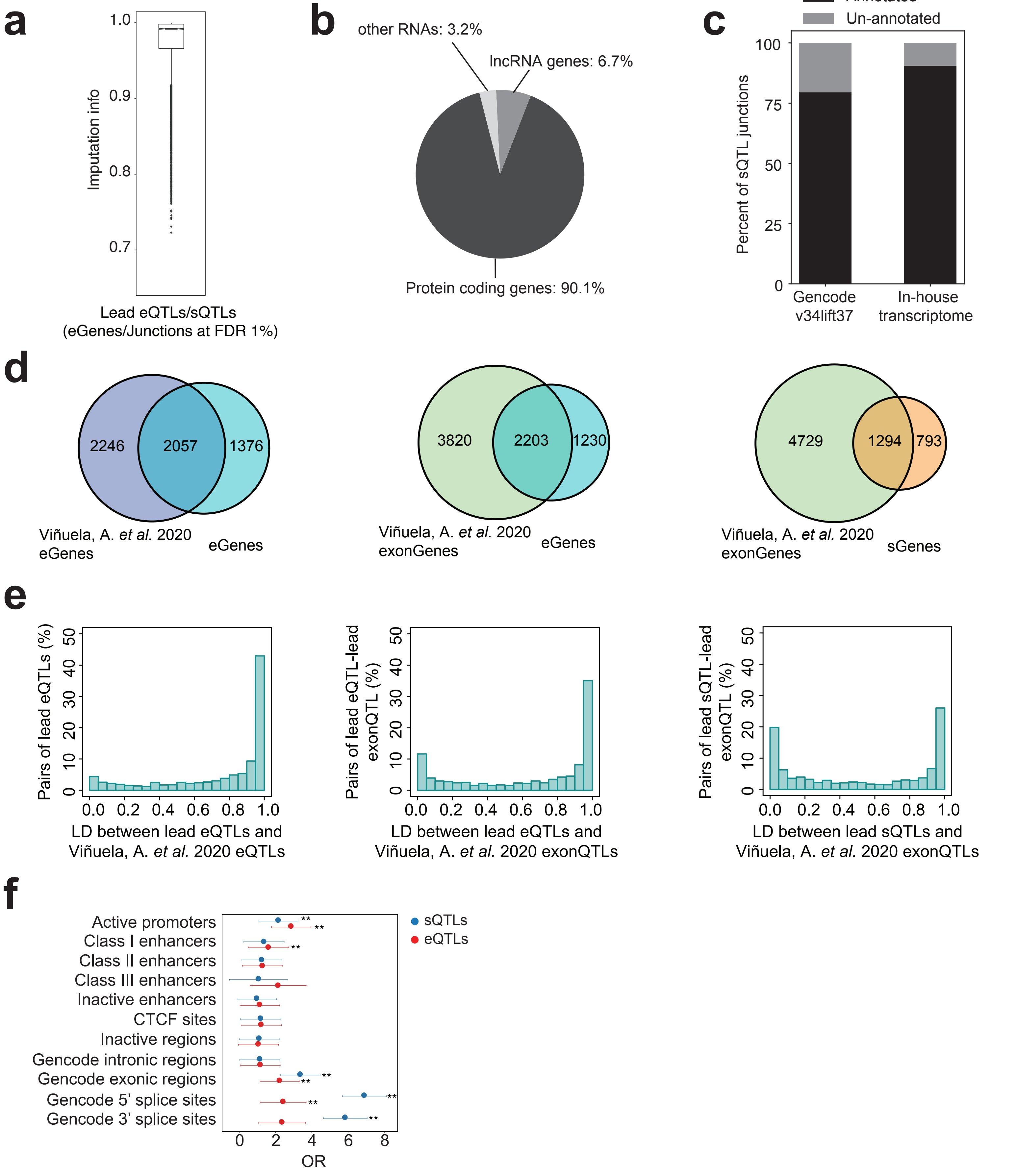

Figure S2. Characterization of sQTLs in human islets. A Imputation information score calculated using QCTOOL for lead eQTLs and sQTLs identified at FDR ≤1%. **B** Distribution of sQTL junctions among protein-coding, lncRNAs and other RNAs. **C** Percentage of sQTL junctions successfully annotated using GENCODE or unpublished in-house transcriptome annotations. **D** Venn-diagrams for the overlap between eGenes and sGenes identified in the current study vs. eGenes and exon-QTL genes identified by the InsPIRE consortium. **E** Distribution of linkage disequilibrium (r^2) values between the lead eQTL or sQTL in our study and the lead eQTL or exonQTL in the InsPIRE consortium in shared QTL genes. **F** Enrichment of sQTL and eQTL variants in different functional genomic annotations using GARFIELD (QTL p-value threshold = $5x10^{-5}$). The x-axis shows the GARFIELD odds ratio (OR) of the enrichment at each functional annotation. Whiskers show the 95% CI of the GARFIELD OR and two asterisks indicate significant enrichments after considering the number of effective annotations, the p-value thresholds used and the molecular QTL traits.

Fig. S3

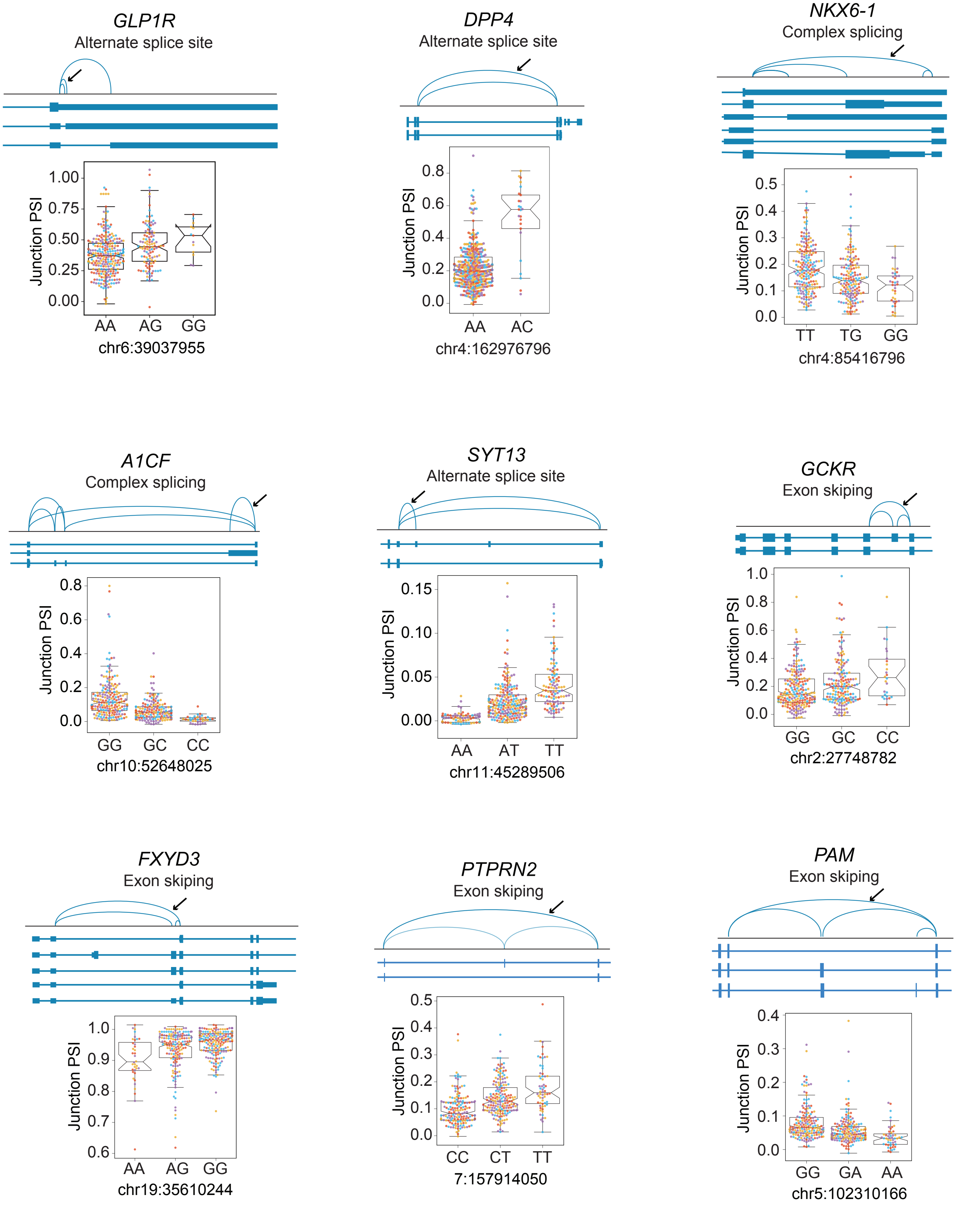

Figure S3. sQTLs at selected genes with central roles in islet cellular identity and diabetes pathophysiology. Boxplot representations show the junction percent spliced in (PSI) values for the lead sQTL genotypes. The IQR of the junction PSI distribution is represented as boxes and whiskers are 1.5 x IQR. Individual PSI junction values are colored according to batches, as indicated in **Fig. 1a**. All sQTL effects are significant at FDR ≤ 1%, see **Additional file 3: Table S2.**

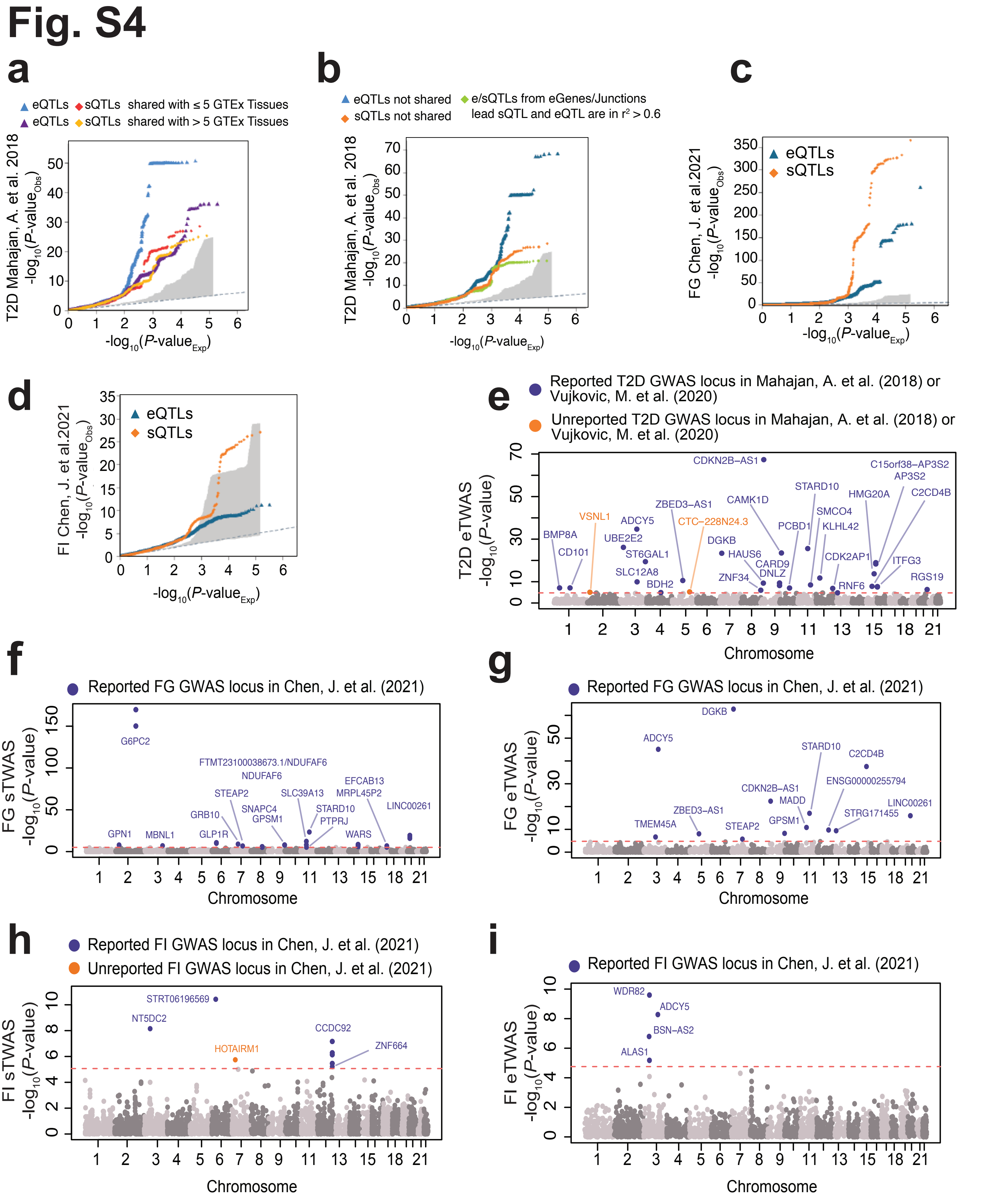

Figure S4. Role of islet sQTLs in T2D and related traits. A-D Quantile-quantile (QQ) plot representations show $-\log_{10}$ association p-values (y-axis) for sQTLs (orange) and eQTLs (blue), which are represented against the p-values under the null hypothesis (x-axis). The grey shaded area shows observed $-\log_{10}$ p-values across 1000 random samplings of control sets of variants, with the same size as islet sQTLs. **A** Inflation of T2D association p-values across eQTLs and sQTLs shared with either less or more than 5 GTEx tissues. **B** Inflation of T2D association p-values for islet eQTLs and sQTLs that are independent vs. those in which a lead eQTL and sQTL are in $r^2 > 0.6$. **C-D** Genomic inflation in fasting glycemia and fasting insulin association p-values for islet sQTLs and eQTLs. **E-I** Manhattan plots showing splicing (sTWAS) and gene expression (eTWAS) associations with T2D and variation of glycemic traits. TWAS p-values are shown in the y-axis in the $-\log_{10}$ scale. Significant TWAS associations in known GWAS loci are colored in purple while orange dots highlight significant TWAS associations in genomic regions that were not previously reported. FG = fasting glucose, FI = fasting insulin.

Fig. S5

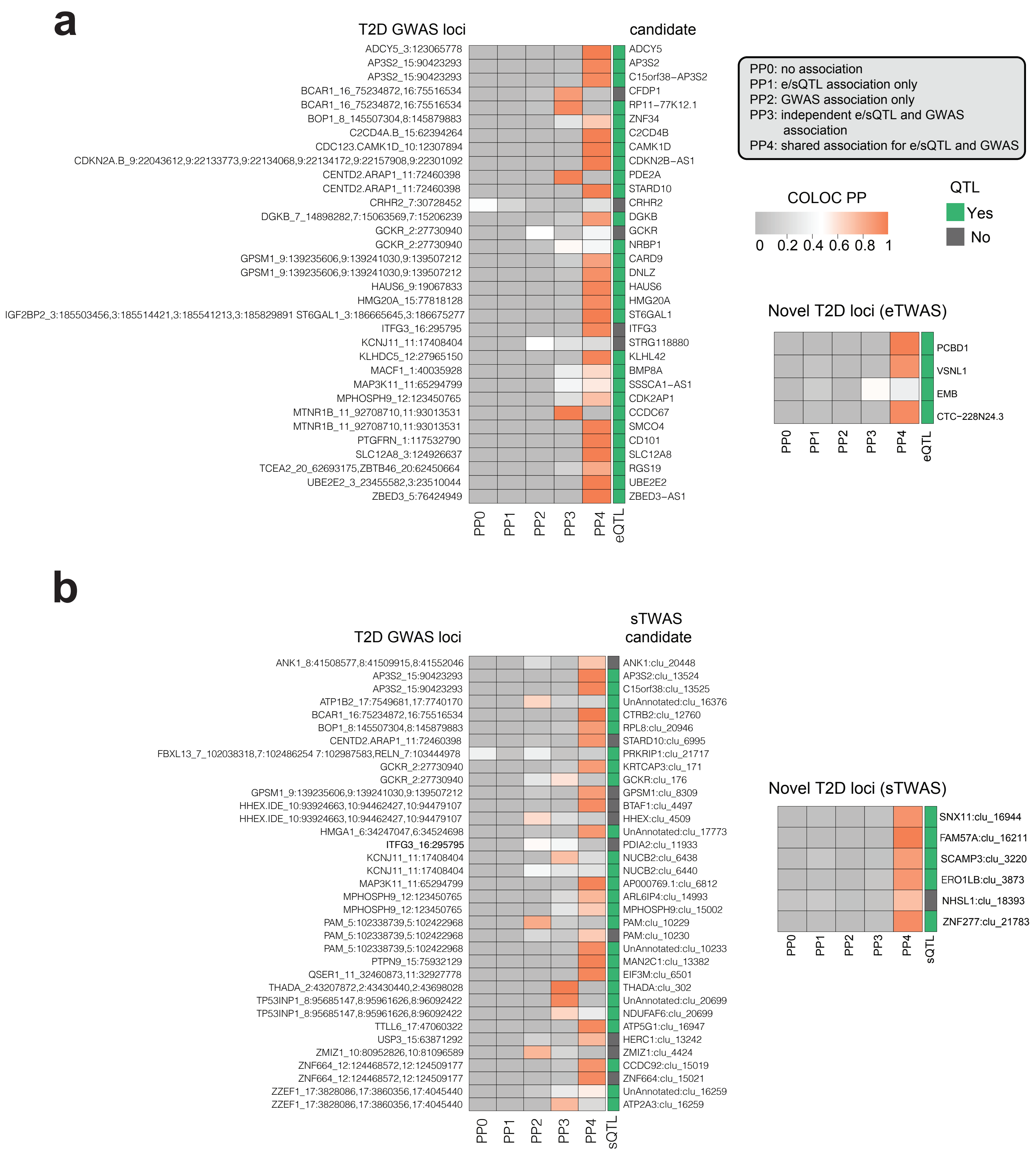

Figure S5. Colocalization posterior probabilities for TWAS associations. A-B Heatmap representations show colocalization posterior probabilities from COLOC for **A** eTWAS and **B** sTWAS associations. Separate heatmaps are provided for known and novel T2D loci identified by TWAS. Heatmap color indicates five possible colocalization posterior probability values (PP0-PP4, see inset). TWAS-prioritized candidate genes that are also significant sQTL or eQTLs in human islets are highlighted in green.

Fig. S6

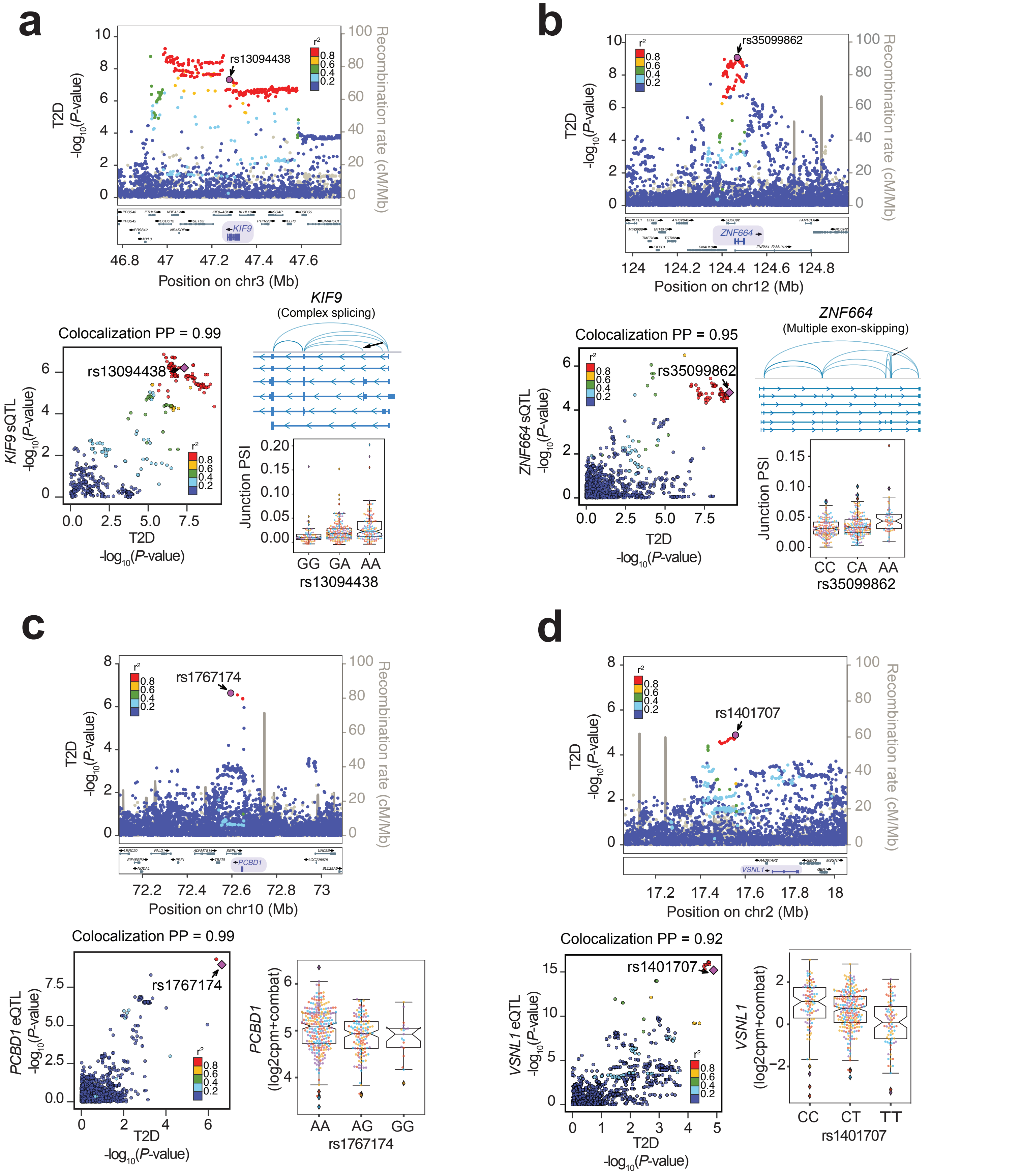

Figure S6. Regional signal plots for a sQTL colocalization, and sTWAS and eTWAS associations mediating T2D risk. A *KIF9* sQTLs (significant at FDR ≤ 1%, see **Additional file 3: Table S2**) show high colocalization support at a known T2D GWAS locus. **B** sTWAS association for T2D risk in the *ZNF664* gene (lead sQTL q-value = 0.014). **C-D** eTWAS associations mediated by *PCBD1* expression in a known T2D locus, or for *VSNL1* in a previously unreported T2D association locus. eQTLs at *PCBD1* and *VSNL1* are significant at FDR ≤ 1%, see **Additional file 2: Table S1**. For all panels, LocusZoom representations show $-log_{10}$ T2D association p-values across the genomic position in hg19 genome build in the x-axis. Variants are colored according to the LD correlation (r^2) with the lead variant of the sTWAS association (purple). LocusCompare scatterplots show correlations between e/sQTL and T2D GWAS association p-values ($-log_{10}$ scale), colored according LD correlations (r^2) with the lead GWAS variant from TWAS (purple) as the color scheme. Boxplots show PSI junction values or normalized gene expression values for the lead TWAS association variant genotypes. Boxplots are colored by batches, as indicated in **Fig. 1a**.

Fig. S7

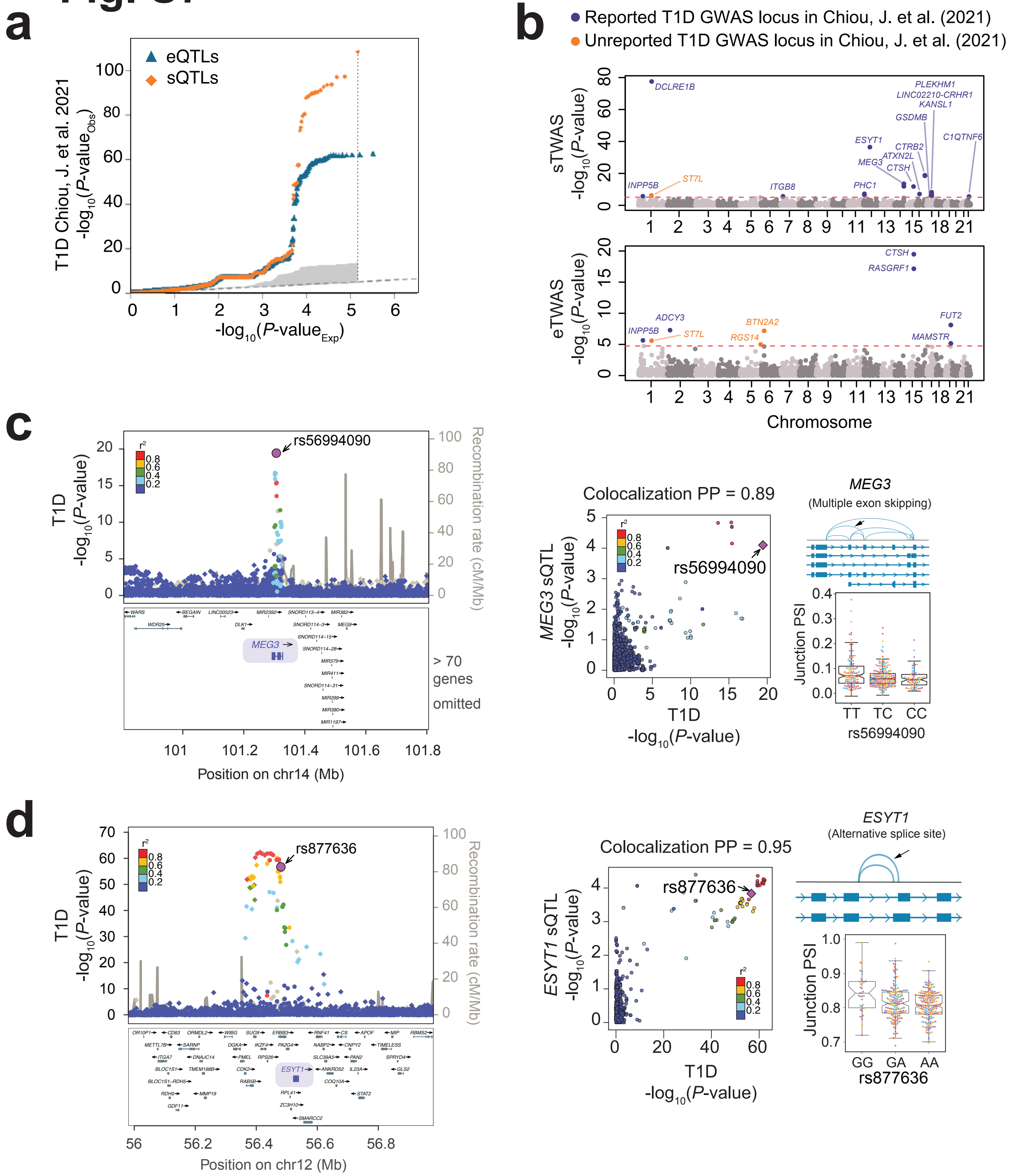

Figure S7. Islet sQTLs and eQTLs inform T1D risk alleles. A QQ plot representation compares $-\log_{10}$ T1D association p-values (y-axis) for sQTLs (orange) and eQTLs (blue) against the p-values under the null hypothesis (x-axis). The grey shaded area defines observed $-\log_{10}$ T1D association p-values across 1000 random samplings of control sets of variants, with the same size as islet sQTLs. **B** Manhattan plots show splicing (sTWAS) and gene expression (eTWAS) associations with T1D susceptibility. The y-axis shows TWAS p-values in the $-\log_{10}$ scale. Significant TWAS associations in known T1D GWAS loci are colored in purple while orange dots highlight significant TWAS associations in genomic regions that were not previously reported. T1D = type 1 diabetes. **C-D** Regional plots at *DLK1/MEG3* and *IKZF4* known T1D GWAS loci, which show sTWAS associations at *MEG3* and *ESYT1* genes, respectively. The splicing QTL effects at *MEG3* and *ESYT1* are significant at FDR ≤ 1%, see **Additional file 3: Table S2**. Locuszoom representations show $-\log_{10}$ T1D association p-values (y-axis) at genomic positions in the hg19 genome build (x-axis), colored according LD correlations (r^2) with the lead sTWAS association (purple). Locuscompare scatterplots compare sQTL and T1D GWAS association p-values ($-\log_{10}$ scale), colored as in the locuszoom representations. Boxplots show individual PSI junction values according to the lead TWAS association variant genotypes, colored as indicated in **Fig. 1a**.

Fig. S8

a

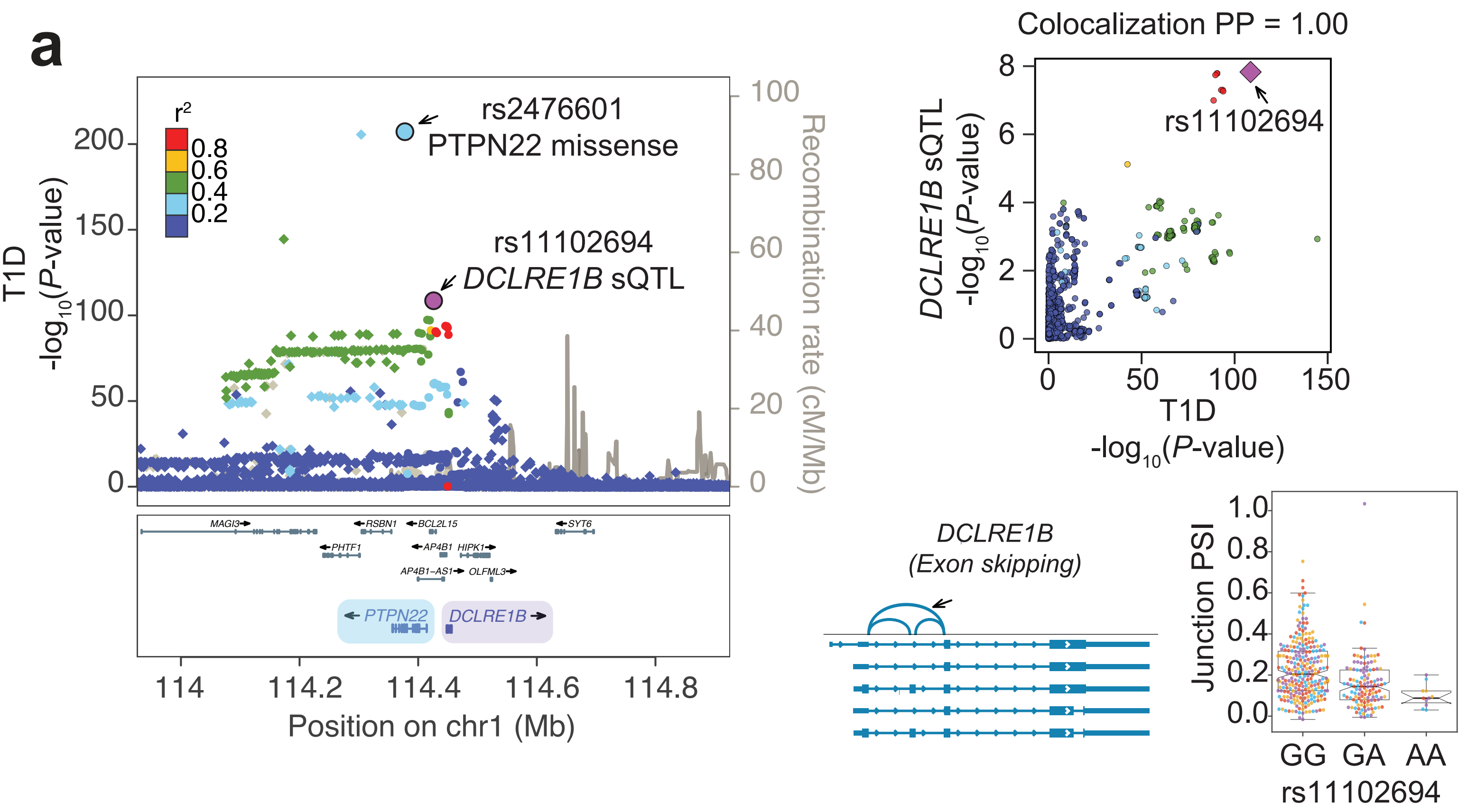

b

snp id	variant type	effect allele	p-value	OR	Conditioning on rs2476601 p-value	Conditioning on rs2476601 OR	Conditioning on rs11102694 p-value	Conditioning on rs11102694 OR
rs2476601	*PTPN22* missense	G	5.91×10^{-208}	0.53	-	-	1.32×10^{-68}	0.69
rs11102694	*DCLRE1B* sQTL	A	2.78×10^{-109}	1.46	1.17×10^{-4}	1.07	-	-

* LD calculations using 503 1000 Genomes Phase 3 individuals of European descend.

c

snp id	variant type	effect allele	p-value	OR	Joint effect model p-value	Joint effect model OR
rs2476601	*PTPN22* missense	G	5.91×10^{-208}	0.53	1.73×10^{-101}	0.55
rs11102694	*DCLRE1B* sQTL	A	2.78×10^{-109}	1.46	2.71×10^{-2}	1.05

* LD calculations using 381,380 UKBB individuals.

Figure S8. TWAS identifies a novel association between an exon-skipping event at *DCLRE1B* with T1D risk. A LocusZoom representation shows -$\log_{10}$ T1D association p-values across the *DCLRE1B* genomic position in the hg19 genome build. Variants are colored according to the LD correlation (r^2) with the lead GWAS variant from sTWAS results. LocusCompare scatter plot shows correlations between sQTL and T1D GWAS association p-values (-$\log_{10}$ scale), colored according to the LD patterns, as in the locuszoom representation. Boxplot shows PSI junction values for the lead TWAS association variant genotypes, colored as indicated in **Fig. 1a**. The splicing QTL effect at *DCLRE1B* is significant at FDR ≤ 1%, see **Additional file 3: Table S2**. **B-C** Interpretation of the rs11102694 *DCLRE1B* sQTL T1D association considering one of the strongest T1D signals in the same locus, the rs2476601 *PTPN22* missense variant. **B** Single conditional analysis with GCTA including each variant (rs11102694 *DCLRE1B* sQTL and rs2476601 *PTPN22* missense variant) at a time. Individuals with European descend from 1000 Genomes Phase 3 were used for LD calculations. **C** Joint analysis of SNP effects in GCTA using a fraction of unrelated UK Biobank individuals of white European origin for LD calculations. Summary statistics for each variant association are showed after single-variant conditional analysis or in the joint SNP effects model.

Fig. S9

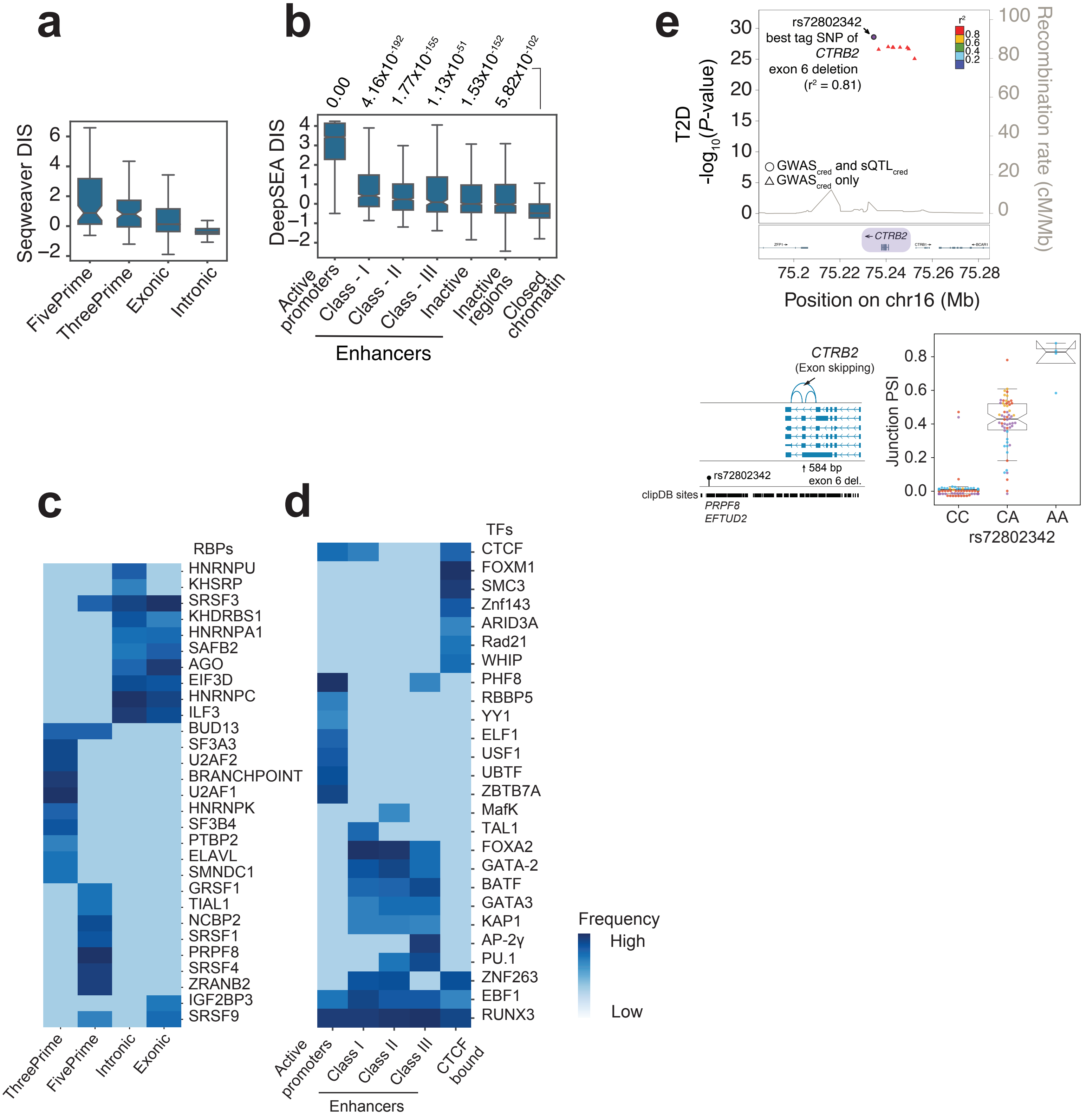

Figure S9. Functional annotation of fine-mapped sQTL and eQTL variants. A Distribution of disease impact scores (DIS) trained using Seqweaver in fine-mapped (credible set) sQTL variants across relevant functional annotations. **B** DIS values trained using the DeepSEA model in eQTL credible set variants located in human islet active promoters, enhancers, accessible chromatin regions lacking active modifications (*inactive* regions), and regions with *closed* (inaccessible) chromatin. **C-D** Disruption of DNA- and RNA-binding protein sequences by eQTLs and sQTLs. sQTL credible set variants show increased disruption of largely expected sets of RBP sites according to their genomic location. Similarly, eQTL credible set variants show increased frequency at different TF binding sites according to their genomic location in different cis-regulatory elements. The frequency indicates the ranking of RBP/TF based on the number of credible set variants impacting them at each category. Top ranked RBP/TF within each category are shown in dark blue. **E** Fine-mapping T2D associations and *CTRB2* sQTLs in the *BCAR1* T2D, T1D and PDAC locus. Regional signal LocusZoom plot shows T2D association p-values (in the $-\log_{10}$ scale) and locations in the hg19 genome build. Credible set variants for GWAS and sQTLs are represented as circles, and other GWAS credible set variants as triangles. Variants are colored according to the LD (r^2) with rs72802342, the lead and best candidate causal GWAS variant (in purple). This candidate GWAS and sQTL causal variant (rs72802342) is the best tag SNP of a 584bp deletion of exon 6 in *CTRB2* (r^2=0.81). The bottom inset depicts the alternative splicing event, along with the candidate causal sQTL variant, and clipDB RBP binding sites where it is located. Boxplots are represented as described before.
